# Placental Kisspeptins Differentially Modulate Vital Parameters of Estrogen Receptor-Positive and -Negative Breast Cancer Cells

**DOI:** 10.1371/journal.pone.0153684

**Published:** 2016-04-21

**Authors:** Zahra Rasoulzadeh, Roya Ghods, Tohid Kazemi, Ebrahim Mirzadegan, Nassim Ghaffari-Tabrizi-Wizsy, Simin Rezania, Somaieh Kazemnejad, Soheila Arefi, Jamileh Ghasemi, Sedigheh Vafaei, Ahmad-Reza Mahmoudi, Amir-Hassan Zarnani

**Affiliations:** 1 Department of Immunology, Tabriz University of Medical Sciences, Tabriz, 5165683146, Iran; 2 Immunology Research Center, Tabriz University of Medical Sciences, Tabriz, 5165683146, Iran; 3 Department of Molecular Medicine, Faculty of Advanced Technologies in Medicine, Iran University of Medical Sciences, IUMS, Tehran, 1449614535, Iran; 4 Oncopathology Research Center, Iran University of Medical Sciences, Tehran, 1449614535, Iran; 5 Immunobiology Research Center, Avicenna Research Institute, ACECR, Tehran, 1177–19615, Iran; 6 SFL Chicken CAM Lab, Institute of Pathophysiology and Immunology, Medical University of Graz, Graz, 8010, Austria; 7 Institute of Biophysics, Medical University of Graz, Graz, 8010, Austria; 8 Reproductive Biotechnology Research Center, Avicenna Research Institute, ACECR, Tehran, 1177–19615, Iran; 9 Nanobiotechnology Research Center, Avicenna Research Institute, ACECR, Tehran, 1177–19615, Iran; 10 Monoclonal Antibody Research Center, Avicenna Research Institute, ACECR, Tehran, 1177–19615, Iran; 11 Immunology Research Center, Iran University of Medical Sciences, Tehran, 81746–73461, Iran; Sudbury Regional Hospital, CANADA

## Abstract

Kisspeptins (KPs) are major regulators of trophoblast and cancer invasion. Thus far, limited and conflicting data are available on KP-mediated modulation of breast cancer (BC) metastasis; mostly based on synthetic KP-10, the most active fragment of KP. Here, we report for the first time comprehensive functional effects of term placental KPs on proliferation, adhesion, Matrigel invasion, motility, MMP activity and pro-inflammatory cytokine production in MDA-MB-231 (estrogen receptor-negative) and MCF-7 (estrogen receptor-positive). KPs were expressed at high level by term placental syncytiotrophoblasts and released in soluble form. Placental explant conditioned medium containing KPs (CM) significantly reduced proliferation of both cell types compared to CM without (w/o) KP (CM-w/o KP) in a dose- and time-dependent manner. In MDA-MB-231 cells, placental KPs significantly reduced adhesive properties, while increased MMP9 and MMP2 activity and stimulated invasion. Increased invasiveness of MDA-MB-231 cells after CM treatment was inhibited by KP receptor antagonist, P-234. CM significantly reduced motility of MCF-7 cells at all time points (2–30 hr), while it stimulated motility of MDA-MB-231 cells. These effects were reversed by P-234. Co-treatment with selective ER modulators, Tamoxifen and Raloxifene, inhibited the effect of CM on motility of MCF-7 cells. The level of IL-6 in supernatant of MCF-7 cells treated with CM was higher compared to those treated with CM-w/o KP. Both cell types produced more IL-8 after treatment with CM compared to those treated with CM-w/o KP. Taken together, our observations suggest that placental KPs differentially modulate vital parameters of estrogen receptor-positive and -negative BC cells possibly through modulation of pro-inflammatory cytokine production.

## Introduction

Invasion of placental trophoblast cells into the maternal uterine decidua and vasculature is the hallmark of haemochorial placentation. During placental development and differentiation, extravillous trophoblasts (EVT) undergo substantial molecular modifications exemplified by the over expression of matrix metalloproteinases (MMPs) and gain the ability to invade extracellular matrix. From biological point of view, trophoblast invasion shares common features with tumor invasion and metastasis with similar molecular machinery and mechanisms. In both trophoblast and cancer cells, acquisition of invasive phenotype is accompanied by several coordinated events including repression of specific cell adhesion molecules, augmentation of cell motility, expression of MMPs and proto-oncogene products and establishment of immunosuppressive environmental conditions[1–3].

In spite of aforesaid similarities and contrary to tumor cells, trophoblast invasion is under tight spatiotemporal control[4]. Such controlled invasion is of crucial importance for maternal health and growing fetus development. In this context, several autocrine and paracrine regulatory systems work in concert to limit trophoblast invasion. Gonadotropin releasing hormone (GnRH) and tumor necrosis factor (TNF)-alpha are among the factors derived from placenta and exert considerable inhibitory action on trophoblast migration and invasion[5, 6].

Kisspeptins (KPs) are major regulator of trophoblast invasion[7]. This family of regulatory peptides is originated from the *KISS1* gene translation product following proteolytic processing. The largest form, also known as KISS1, consists of 145 amino acids[8], which are processed by proteolytic enzymes to shorter fragments of 54 (KP-54; metastin), 14 (KP-14), 13 (KP-13) or 10 (KP-10) amino acids. The common feature of all KPs is a C-terminal ten residue peptide (KP-10) necessary for receptor activation[7]. KPs exert their regulatory actions after binding to their cognate receptor, KISS1R[9]. Among all KPs, KP-10 has the highest potency to bind KISS1R and to trigger downstream signaling pathway[10]. In trophoblasts of early placenta, it was shown that only KP-10 was able to increase intracellular Ca^2+^[7, 11]. KISS1R activation following KP binding results in activation of phospholipase C, phosphatidylinositol turnover, calcium mobilization, and stimulation of extracellular signal-regulated kinase-1 and -2 (ERK1 and ERK2) and mitogen-activated protein (MAPKs)[8–10].

In addition to placenta, KISS1 transcript is also expressed at high levels in other tissues including brain, breast, pancreas, testis, liver, heart and small intestine. In line with its autocrine action, *KISS1R* mRNA displays similar tissue distribution as *KISS1*transcript. KISS1/KISS1R system has also been shown to be active in many cancer cell types with profound anti-metastatic action[12, 13].

In case of breast cancer, the data on potential action of KISS1 on invasiveness and metastasis is rather controversial. As with anti-metastatic action in melanoma cell lines C8161 and MelJuSo, initially it was shown that KISS1 acts as a metastasis suppressor in human breast cancer cells, MDA-MB-435[14]. More recent studies highlighted the positive correlation of KISS1/KISS1R system with breast cancer progression and patient prognosis[15]. Moreover, expression of KISS1 was higher in patients who had died from breast cancer than those who had survived[16]. Indeed, higher expression levels of KISS1 and KISS1R was found to be positively correlated with shorter relapse-free survival[15]. The clue for such discrepancy was proposed by Cvetkovic´ *et al*. who clearly showed that it is the ER status of mammary cells that dictates KP responsiveness. Besides the effect of endogenous KISS1 expression by breast cancer cells on their own vital parameters including proliferation and invasion, exogenous KISS1 may also alter invasive capacity of tumor cells [17]. It is proposed that placental factors facilitate motility of some breast cancer cell lines and thus contribute to the advanced breast cancer found during pregnancy [18].

Thus far, almost all the data on potential action of KPs over vital parameters of cancer cells has been derived from the experiments in which synthetic KP-10 has been used. To have a better understanding of functional activity of the native molecule, one may need to know how cancerous activity of malignant cells is affected by the KPs released from its physiological sources such as placenta. To this end, we embarked on comprehensive analyses of the potential effects of placental KPs and synthetic KP-10 on proliferation, adhesion, invasion, migration, MMP activity and pro-inflammatory cytokine production in ER^-^ (MDA-MB-231) and ER^+^ (MCF-7) breast cancer cells.

## Materials and Methods

### Placental unit collection

All experimental procedures listed in this research work was conducted after being approved by ethical committee of Avicenna Research Institute (92.1630) in accordance with Helsinki guidelines and regulations and all participants signed a written consent form before enrolment in this study. From eleven healthy women (22 to 32 years) with uncomplicated term pregnancies undergoing elective cesarean, placentas were obtained, suspended in culture medium and immediately transferred to the laboratory in a cold chain under sterile condition. Before cesarean, all women were examined for such blood born viral infections as HIV, HBV and HCV and checked to make sure they did not have vaginal infection.

### Isolation of term placental cytotrophoblast cells

All experimental procedures were carried out under sterile conditions. Isolation of cytotrophoblasts was performed according to the protocol published elsewhere[19]. In brief, placentas were placed in a sterile stainless steel dish under biological hood with maternal side of the placenta facing up. About 50 g of villous tissue was carefully removed using scissors and forceps and placed in a sterile beaker. Tissue fragments were rinsed several times by phosphate buffered saline to remove blood clots. Tissues were then transferred to a 150 mm culture plate and minced finely with scissors. Obtained small fragments were rinsed again over cell dissociation sieve and digested in three successive steps, each for 15–20 min, at 37°C in an enzymatic cocktail containing DNase (Roche, Germany) and Trypsin (Sigma, USA). After collecting the cells from all three digestion stages, cell suspensions were pooled and filtered using a 100 μm nylon cell strainer. The resulting cells were layered over preformed Percoll (GE Healthcare, Sweden) gradient (5–70%). Upper diffuse band (≅1.046–1.065) was collected as cytotrophoblast cells and washed three times with RPMI.

### Immunofluorescent staining of placental tissues, purified cytotrophoblasts and breast cancer cells

Detection of KPs and cytokeratin 7 (CK7) was performed by Immunofluorescent staining[20]. In brief, small fragments of placental tissue were fixed for 48 hr in 10% formalin, embedded in paraffin and cut into 4 μm sections. Slides were then deparaffinized in xylene, hydrated in increasing grade of ethanol and rinsed several times in tap water. Antigen retrieval was performed in 95°C heated Tris-EDTA (10mM) buffer for 30 min. After blocking of non-specific binding sites by 5% non-immune sheep serum, 1:1000 dilution of purified rabbit anti-human KP-54 was applied to the slides for 90 min at room temperature (RT). Slides were then washed in PBS containing 1% bovine serum albumin (PBS-BSA) three times and incubated in Dylight 488-goat anti-rabbit (1:800) for 90 minutes at RT in the dark. All subsequent incubation steps were done in the dark. Washing step was proceeded as above followed by incubation with 1:800 dilution of mouse anti-human CK7 for 30 min. After washing, slides were treated with CY3- goat anti-mouse (1:800) for 45 min (all antibodies were generously gifted by Department of Cell Biology, Histology and Embryology, Medical University of Graz). Nuclei were visualized by diamidino-2-phenylindole (DAPI) staining for 5 min. At the final step, slides were washed, mounted with PBS-glycerol (50% v/v) and inspected under epifluorescent microscope equipped with DP70 CCD camera (Olympus, Japan). Single color KPs staining of purified cytotrophoblasts and breast cancer cells was performed as above with some modifications[21]. Briefly, cells were cytospinned on poly-L-lysine-coated slides, dried at RT for 15 min and fixed in ice-cold pure acetone for 5 min. After three washes, primary and secondary antibodies were applied at aforesaid concentrations and slides were processed as above. In negative controls, primary antibodies were substituted by non-immune sera from rabbit or mouse.

### Culture of placental villous tissue explants

With maternal side of placenta facing up, 3–5 mm fragments of cotyledons were dissected using forceps and scissors and placed in petri dish containing PBS. The villous tissue was then carefully isolated and cut into similar fragments of about 5 mg and four pieces were placed in each well of 24-well plates coated with matrigel (BD Biosciences, USA). Plate was incubated at 37°C CO2 incubator for 3 hr for initial attachment. After then, 300 μl serum-free DMEM-Ham′s F12 (PAA, Germany) supplemented with 2 mM L-glutamine, 100 IU/mL penicillin and 100 μg/mL streptomycin was slowly added to each well and incubation was continued for further 2–72 hr. At different time points from one to72 hr, culture supernatants were collected and examined for the content of KPs released from placental explants by Western blotting as described below. Based on the results, all subsequent culture supernatants were collected 4 hr after addition of culture medium, centrifuged and stored at -80°C as conditioned medium (CM).

### Cell Culture

Human breast carcinoma cells, MDA-MB-231 and MCF-7 cell lines were purchased from national cell bank of Iran (NCBI, Tehran, Iran) and cultured in DMEM (Gibco, USA) supplemented with 5% (v/v) fetal bovine serum (FBS) (Gibco), 2 mM L-glutamine, 100 IU/mL penicillin and 100 μg/mL streptomycin. Cells were maintained in culture not more than three passages.

### Reverse transcriptase polymerase chain reaction (RT-PCR)

Total RNA from MDA-MB-231 and MCF-7 cells was extracted using RNeasy kit (Thermo Scientific, Lithuania) according to the manufacturer’s instructions. RNA concentration was measured at 260/280 nm and its quality was evaluated by gel electrophoresis. cDNA synthesis and RT-PCR was performed as described elsewhere[22]. Briefly, purified RNA was heated to 65°C and immediately cooled on ice. cDNA Mix containing 2 μg RNA, 5× buffer (Fermentase, Vilnius, Lithuania), 2 mM dNTP mix (Roche, Penzberg, Germany), 2 mM Random hexamer (Cybergene, Stockholm, Sweden), and 20 U⁄mL RT M-MuLV (Fermentase) was incubated for 60 min at 42°C. One μL cDNA and 1 μL of each pair of primers equivalent to the final concentrations of 0.4 pM for β-actin (as loading control) and 0.2 pM for KISS1, KISS1R, ERα and ERβ were added to 12.5 μL of ready to use master mix (Ampliqon, Denmark). Sequence, amplicon size and Tm of each primer set for KISS1, KISS1R, ERα, ERβ and β-actin have been summarized in Table 1. For all PCR programs, temperatures of 94°C and 72°C were applied for denaturation and extension for 30 seconds followed by final extension for 7 min. PCR was performed 30 (for β-actin) or 35 cycles (for KISS1, KISS1R, ERα and ERβ). Tubes lacking template cDNA or reverse transcriptase (no amplification control) were included as negative controls.

**Table 1:** Sequence and annealing temperatures of the primers used in this study.

	Forward (5′–3′)	Reverse (5′–3′)	Annealing Temp(°C)
*KISS1*	TGTACAACCTGCTGGCGCTG	CCAGTTGTAGTTCGGCAGGT	62
*GRP54*	CACTTTGGGGAGCCATTAGA	CCACTGCTCCCTGGCTTCTG	63.2
*ER-α*	AGACATGAGAGCTGCCAACC	GCCAGGCACATTCTAGAAGG	63
*ER-b*	TCACATCTGTATGCGGAACC	CGTAACACTTCCGAAGTCGG	63
*β- actin*	AGCCTCGCCTTTGCCGA	CTGGTGCCTGGGGCG	60

### Immunoprecipitation of kisspeptins from supernatant of cultured placental explants

Isolation of KPs from placental explants culture supernatants was performed by Dynabeads M-280 Tosylactivated (Invitrogen, USA) according the manufacturer’s recommendations. In brief, magnetic beads were washed and coupled with 100 μg anti-KP-54 for 22 hr at 37°C. After successive washing steps of beads, 1 mL placental explant supernatant was added to the ligand-coupled beads for 90 min. Supernatant was removed on magnet and immediately frozen at -80°C as KP-free conditioned medium (CM-w/o KP).

### SDS-PAGE and Western blotting

The presence of KPs in placenta tissues, supernatants collected from placental explants and MDA-MB-231 and MCF-7 cell cultures were assessed by Western blotting according to the procedure described elsewhere[23]. In brief, tissue and cell lysates were prepared using RIPA lysis buffer (Santa Cruz, USA). Lysates were cleared by centrifugation at 12000 g for 15 min and their protein contents were measured by BCA protein assay kit (Pierce, USA) per manufacturer’s recommendation. Placental explants supernatants were processed without any manipulation. Proteins in lysates or supernatants were fractioned electrophoretically by 15% SDS-PAGE under reducing condition and blotted on a nitrocellulose membrane (Millipore, USA). The membrane was blocked in 5% non-fat dry milk (Merck, Germany) containing 0.05% Tween 20 overnight at 4°C. After washing steps, the membrane was incubated with anti-KP-54 diluted 1:7500 in 5% non-fat dry milk for 90 min at RT. Membrane was then washed and probed with horseradish peroxidase (HRP)-conjugated sheep anti-rabbit Ig (1:5000)(Sina Biotech, Tehran, Iran) for 1 hr at RT followed by washing steps. Signals were visualized by ECL Western Blotting Detection Reagents (GE Healthcare, Sweden) according to the manufacturer’s instruction. For blotting of synthetic KP-10, 18% SDS-PAGE was used followed by the same procedure mentioned above. Negative control lanes received non-immune rabbit IgG with the same concentration as anti-KP-54 instead of primary antibody.

### Detection of kisspeptins in supernatants of cultured placental explants by ELISA

Wells of microtiter polystyrene strips (Maxisorp, Nunc, Denmark) were coated with 10 μg/mL of anti-KP-54 in a volume of 100 μL, for 2 hr at 37°C. Negative control wells received PBS as coating material. Strips were then washed three times with PBS-Tween (0.05%) (PBS-T) and blocked using PBS containing 2.5% BSA and 10% FBS overnight at 4°C. After washing as above, 100 μL supernatants of cultured placental explants were added to the wells for 90 min at 37°C. Some wells received culture medium as further negative controls. Wells were thoroughly washed with PBS-T and treated with 500 ng/mL biotinylated anti-KP-54 (biotinylation was conducted in ARI) for 30 min at 37°C. Washing step was proceeded as above followed by addition to the wells of 1:12500 dilution of HRP-Streptavidin (Invitrogen, USA) for 30 min at 37°C. After the last washing step, reaction was visualized using TMB chromogen (USB, USA) and optical densities were recorded by an ELISA reader (Anthos 2020, Cambridge, England) at 450 nm.

### Cell proliferation assay

Measurement of cell proliferation was performed as with protocol we published recently with some modifications[24]. Briefly, linearity of measurement was first confirmed by seeding increasing numbers of MDA-MB-231 and MCF-7 cells into the wells of a 96-well plate. Based on the results, cell density of 4×10^3^ cells/well, corresponding to the lower part of linear portion of standard curve, was selected. Cells were seeded at aforesaid density and treated with different media as below: final dilutions of 1:2, 1:4 and 1:8 of CM or CM-w/o KP, 1:2, 1:4 and 1:8 dilutions of CM plus 20 μg/mL anti-KP-54 or the same concentration of normal rabbit IgG, culture medium alone or containing either 20 μg/mL anti-KP-54, normal rabbit IgG or 100 nM KP-10 (Sigma). All treatments were performed in six replicas. Cultures were continued for 24, 48 and 72 hr and the extent of proliferation was measure by Propidium Iodide (PI) fluorometric assay. At each time point, cells were lysed by 0.2% Triton X100 for 30 min on a shaker. DNA content of lysed cells was stained in the dark for 15 min with 3 mM PI (Sigma). Fluorescent intensity of the wells was measured by Victor IIX multilabel plate reader (PerkinElmer, USA) at excitation and emission wavelength of 531 nm 595 nm, respectively.

### Adhesion Assay

Attachment of MDA-MB-231 and MCF-7 cells to fibronectin-coated wells (BD biosciences, USA) was assessed as described recently[25]. Briefly, a predetermined optimal cell number of 2.5×10^4^/well MDA-MB-231 or 5×10^5^/well MCF-7 was seeded into the wells of attachment plates in DMEM containing 5% FBS. Immediately, cells were treated with different media as stated above for cell proliferation assay. All treatments were performed in five replicas. Plates were incubated for 150 min at CO2 incubator. Thereafter, non-adherent cells were removed by gentle washing and adherent cells were fixed with 96% ethanol and stained with 0.1% crystal violet. At the next step and in order to extract crystal violet, cells were lysed with 10% (v/v) acetic acid and optical density of the wells were read using Anthos 2020 ELISA reader at 570 nm.

### Cell Invasion Assay

The protocol for cell invasion assay was adapted from the recent paper by Nikoo *et al*.[26]. Briefly, invasive MDA-MB-231 and non-invasive MCF-7 cells were plated in the Matrigel-coated upper chamber of 24-well invasion plates (8 μm pores) (BD biosciences, USA) at a density of 25000 cells/well. Lower chambers contained DMEM supplemented with 5% FBS as chemoattractant. Cells were treated either with 1:2 dilution of CM or CM-w/o KP. In order to implicate the role of the KISS1R in modulating the process of MDA-MB-231 invasion by placental KPs, some wells were treated with 1 μM KISS1R antagonist, P-234 (PHOENIX PHARMACEUTICALS, USA, a gift from Dr. Mahmoudi, Shahid Beheshti University) three hours before and also during treatment with CM. Some wells were also treated with 100 nM KP-10 or medium without supplement. All treatments we performed in duplicate. Incubation for MDA-MB-231 and MCF-7 cells was continued at a CO2 incubator for 22 hr and 48 hr, respectively. Cells remained in the upper side of the invasion filter were carefully scrapped off by a pre-wet cotton swab and invading cells to the lower surface of the filter were fixed in methanol and stained with 1% crystal violet. Filters were carefully removed and mounted on glass slides. Invading cells were enumerated in 50 random fields using an Olympus BX51 microscope and expressed as percentage of corresponding controls.

### Gelatin zymographic detection of MMP2 and MMP9

MBA-MD-231 and MCF-7 cells cultured in serum-free medium were treated with CM, CM-w/o KP or KP-10 for 24 hr. Untreated cells and cells treated with Phorbol 12-myristate 13-acetate (PMA) served as negative and positive controls, respectively. Following treatments, cell culture supernatants were collected on ice and centrifuged to remove cell debris. No procedure was applied to concentrate supernatants. Protein contents of supernatants were determined by Bradford method. A volume containing equal amount of protein from collected supernatants was mixed with SDS sample buffer and applied without boiling to 10% SDS polyacrylamide gel copolymerized with 1 mg/ml gelatin (Sigma-Aldrich). After electrophoresis, gels were washed with gentle agitation in renaturing buffer (2.5% Triton X-100 in H2O) to remove SDS for 30 min at RT. The gels were then equilibrated in developing buffer (50 mM Tris, 200 mM NaCl, 5 mM CaCl2, pH, 7.5) at RT with gentle agitation for 30 min. After removing the old developing buffer, the gels were incubated in fresh developing buffer at 37°C overnight. The gels were then stained with 0.5% Coomassie Brilliant Blue and destained. The MMP activities were visualized as clear bands against the blue background of the stained gels.

### Wound healing assay for cell motility

Wells of 24-well plated were coated with 10 μg/mL fibronectin (Sigma) and seeded with MDA-MB-231 and MCF-7 cells(50×10^4^ cell/well) in DMEM containing 5% FBS. Cells were allowed to grow to confluency for up to 48 hr and then serum-starved in DMEM-2% FBS, overnight. On the bottom of the each well a line was drawn with a marker pen. Two straight parallel lines of scratch, perpendicular to the marker line, were made through the cells using sterile blue pipette tips followed by gentle washing with warm culture medium and treatment in duplicate with 1:2 dilutions of CM, or CM-w/o KP, 100 nM KP-10 or culture medium all containing 2% FBS. Some wells were pretreated with P-234 as described in invasion assay. To see the effect of estrogen receptor signaling on modulation of cell motility after treatment with CM, some MCF-7 wells were treated with selective ER modulators, Tamoxifen (100 nM) or Raloxifene (1 nM) (both from Iran Hormone, gifted by Dr. Mohamaddi from Avicenna Research Institute) for 24 hr before and during treatment with CM. Negative control wells received vehicle (Methanol for Tamoxifen and DMSO for Raloxifene) with the same concentrations. Cells were allowed to migrate into the scratch for 30 hr and visualized at time intervals of 0, 2, 4, 6, 12, 22 and 30 hr post-scratch. At each time period, four photographs (just below and just above of crossing point of each scratch line with marker line) were captured from each well. Percent of reduction in scratched areas at each time point compared to initial time point was measured and analyzed using Image J software (http://imagej.nih.gov/ij) and plotted versus time. At the end of culture period, all supernatants were collected, centrifuged and stored at -80°C for cytokine assay.

### Cytokine assay

Concentrations of IL-6 and IL-8 in supernatants obtained at the end of wound healing assay were measured by capture ELISA according to the manufacturers’ recommendations (BD biosciences). For each individual cytokine, optical densities were converted to concentration after plotting the corresponding standard curve. The minimal detection limit for IL-6 and IL-8 assay kits was 3.1pg/mL.

### Statistical analysis

Differences in mean values between groups were analyzed with the Mann-Whitney U test for comparison of 2 groups and the Kruskal-Wallis test with a Dunnett’s posthoc test for comparison of 3 or more groups using GraphPad Prism 4 (GraphPad Software, Inc.). Differences were considered significant when p≤0.05.

## Results

### Human term placenta expresses high levels of kisspeptin

**Fig 1:**
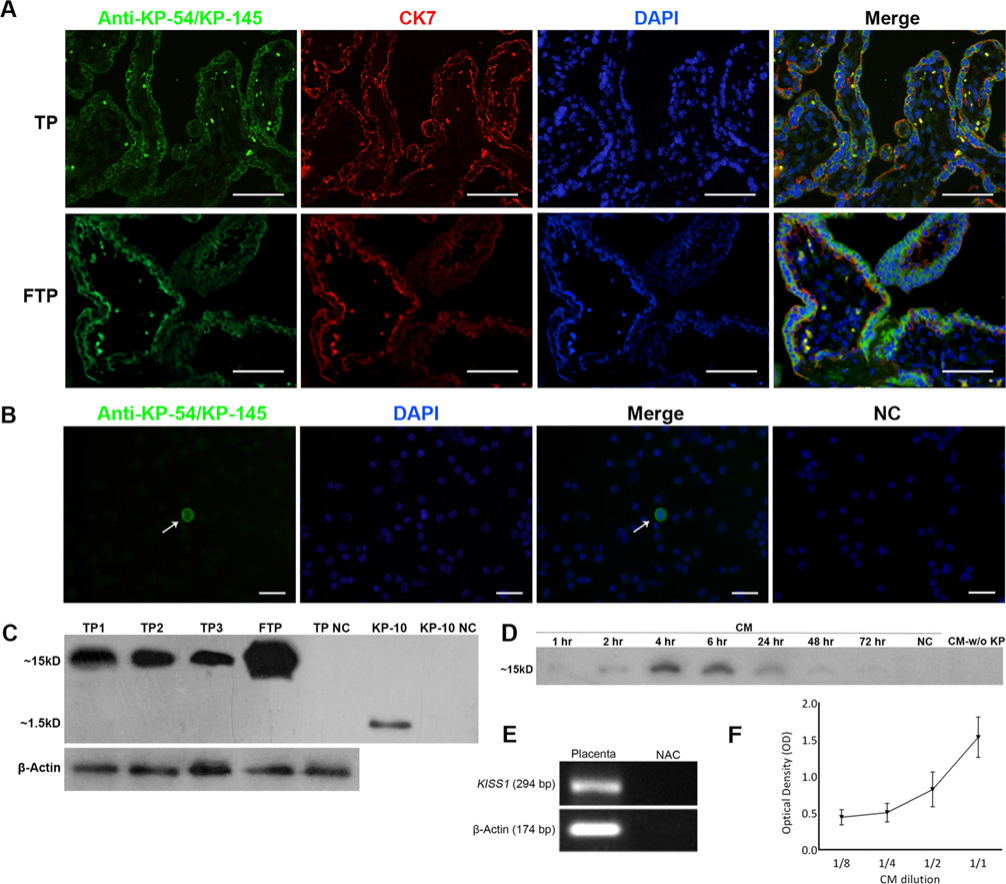
Kisspeptin expression by human term placenta. A) Sections of human term (TP) and first trimester (FTP) placenta were double stained with anti-Kp-54/Kp-145 and anti-CK7, a syncytiotrophoblast (STB) marker. Nuclei were counterstained with DAPI. Kisspeptin was mainly localized to apical and basal surfaces of the STBs. B) Isolated cytotrophoblasts failed to express KISS1. A contaminating positive cell, most probably a STB, has been shown by white arrow. In negative control slides, primary antibody was substituted by non-immune rabbit sera. C) Western blotting of FTP and TP (from three placentas denotes as TP1-3). Lysates were probed with anti-Kp-54/Kp-145 antibody. The antibody produced one prominent band at ~15–16 kDa, which is in good agreement with the calculated MW for Kp-145 (15.391 kDa). With purified Kp-10, anti-Kp-54/Kp-145 produced a single band at about 1.5 kDa, which is in good agreement with the theoretical MW for Kp-10-13. β-actin served as loading control. D) Western blotting of conditioned medium (CM) collected from cultured term placental explants at different time points (1–72 hr). KP was released from placental explants in as early as 1 hr after initiation of culture, peaked at 4–6 hr and then progressively decreased until 72 hr. No KISS1-specific band was observed after KP removal (CM-w/o KP) by immuno precipitation. E) RT-PCR analysis of KISS1expression in total RNA freshly isolated from TP (representative experiment). β-actin served as loading control. No amplification controls (NAC) always were shown to be negative. The presence of KPs in CM was also confirmed by capture ELISA using two fold serially-diluted CMs. The results showed a positive correlation between CM concentration and OD (Fig 1F). Results are representative of 11 term and 3 first trimester placentas. KPs: Kisspeptin, CK7: Cytokeratin 7, TP: Term placenta, FTP: First trimester placenta, NC: Negative control. Scale bar: A; 100 μm, B; 50μm.

In this study, we collected eleven placental units from healthy pregnant women at the time of cesarean delivery and surveyed expression of KPs at both gene and protein levels. To have a rough estimate of how much expression of KPs is down regulated at term pregnancy, when trophoblast invasion is considerably lost, three first trimester placentas from women undergoing elective termination of pregnancy were collected and the expression of KPs was compared with that of term placentas. For immunolocalization of KPs, a rabbit polyclonal antibody raised against KP-54 was used with confirmed immunoreactivity against KP-145 and KP-54[7]. Double color staining of placental sections with anti-KP-54 and CK7 clearly showed that human term syncytiotrophoblasts (STB) expressed KPs at high level. KPs were mainly localized to cell membrane of STBs at both apical and basal surfaces and also to the cell cytoplasm. Notably, KPs were co-localized with CK7, a STB-specific marker. Other cell types including cytotrophoblast cells (CTBs) failed to express KPs (Fig 1A). To confirm this result, CTBs were isolated and individually tested for the expression of KPs. In line with the aforementioned results, no expression of KPs was observed in isolated CTBs (Fig 1B). Although no quantitative measurement was performed, comparative analysis of KPs expression by immunefluorescent staining demonstrated that KPs signal in individual STBs of term placenta was lower compared to that of first trimester placenta (Fig 1A). Indeed, expression of KPs in term placenta was found to be of patchy pattern in which only some areas were positive, while expression pattern in first trimester was more diffuse. We showed here using Western blotting that anti-KP-54 antibody was also able to recognize purified KP-10, albeit with lower reactivity. In this case, a single band of about 1.5 KDa was observed which is in good agreement with the theoretical MW for KP-10, 13 or 14 (Fig 1C). Western blot analysis showed that human term placenta expresses KP-145 (MW = 15.4 KDa) (Fig 1C). First trimester placenta expressed higher levels of KP confirming the results mentioned above (Fig 1C). Placental expression of *KISS1* was checked by RT-PCR. Results of this experiment clearly showed term human placenta expresses *KISS1* transcript (Fig 1E).

### Term placental kisspeptins are released in soluble form

In order to investigate whether or not term placental KPs are released in soluble form, placental explants were cultured and the presence of KPs in the conditioned media was monitored at different time intervals of 1–72 hr. At all time points examined, specific band of KP-145 was observed by Western blotting. Interestingly, KPs were released from placental explants in as early as 1 hr after initiation of culture. The level of release was increased till 4 hr, and then progressively decreased until 72 hr (Fig 1D). Based on these results, in all subsequent experiments; supernatants of placental cultures were collected 4 hr after explantation. The presence of KPs in CM was also checked by capture ELISA using two fold serially-diluted CMs (from 1:1–1:8) to see whether optical densities (OD) of the wells are titrated as well. As negative control, corresponding CMs without KPs were run in parallel. The results showed a positive correlation between CM concentration and OD confirming that our ELISA system technically works (Fig 1F). To prepare KP-free conditioned medium, KPs was removed from placental explants culture supernatants by immunoprecipitation. No KP-specific band was observed after KP removal indicating high efficacy of purification system (Fig 1D).

### MDA-MB-231 and MCF-7 cells express *KISS1* mRNA but not protein

**Fig 2:**
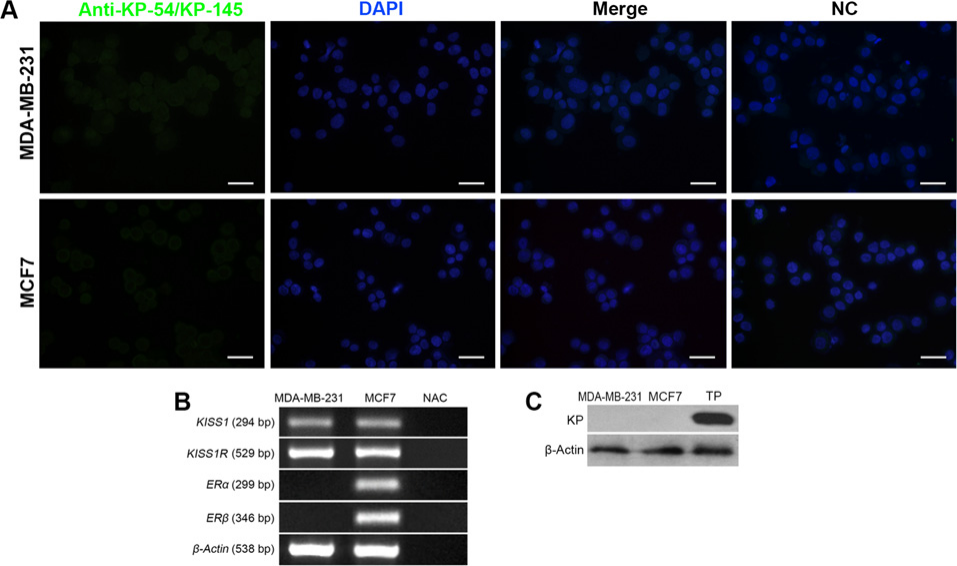
Characterization of ERα, ERβ, KISS1 and KISS1R expression by MDA-MB-231 and MCF-7 breast cancer cells. A) Immunofluorescent staining of MDA-MB-231 and MCF-7 breast cancer cells with anti-Kp-54/Kp-145 showing no KP-specific signal. Nuclei were counterstained with DAPI. In negative control slides, primary antibody was substituted by non-immune rabbit sera. B) RT-PCR of total RNA of MDA-MB-231 and MCF-7 cells. β-actin served as loading control. MDA-MB-231 and MCF-7 cells expressed KISS1 and KISS1R transcripts. No amplification controls (NAC) always were shown to be negative. C) Western blotting of MDA-MB-231 and MCF-7 for KP expression. No KISS-1-specific band was detected. Term placenta (TP) lysate served as positive control. ER: Estrogen receptor, KISS1R: KISS1 receptor, NC: negative control, KP: Kisspeptin. Scale bare: 50μm.

To find out how the cancerous vital parameters of breast cancer cells is affected by placental-derived KPs and having considered the central role of ER expression on biology of most human breast cancer cell types, expression of *ERα*, *ERβ*, *KISS1* and *KISS1R* were investigated by RT-PCR. MCF-7 and MDA-MB-231 cells expressed transcripts of *KISS1* and *KISS1R*. For confirmation, expression of KPs at the protein level was surveyed by two independent experiments, immunofluorescent staining and Western blotting. In spite of positive results for *KISS1* transcript (Fig 2B), KPs were not found to be expressed at protein level in both cell types (Fig 2A and 2C). As expected, *ERα* and *ERβ* were expressed by MCF-7 cells, but MDA-MB-231 cells failed to express these receptors (Fig 2B).

### Placental kisspeptins inhibit proliferation of breast cancer cells

**Fig 3:**
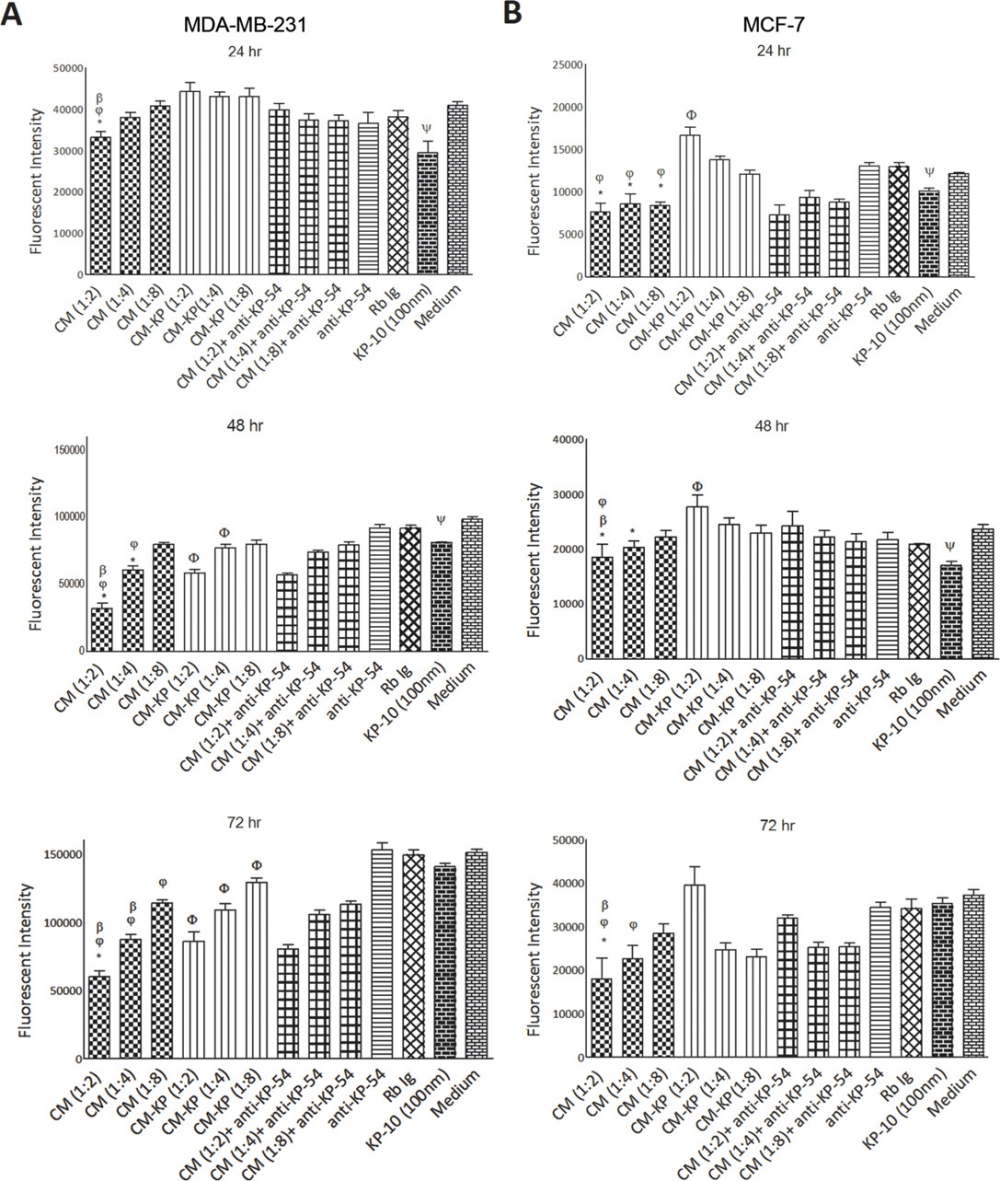
Effect of term placental kisspeptins on proliferation of MDA-MB-231 and MCF-7 breast cancer cells. MDA-MB-231 (A) and MCF7 (B) cells were treated with different media as indicated in the figure for 24, 48 and 72 hr. All treatments were performed in six replicas. The extent of proliferation was measured by Propidium Iodide (PI) fluorometric assay. Significant differences were analyzed by Kruskal-Wallis test with a Dunnett’s posthoc test. Different dilutions from 1:2 to 1:8 were tested. CM: Term placenta conditioned medium containing KP, CM-w/o KP: KP-free CM, anti-KP: anti-Kp-54/Kp-145 antibody, Rb Ig: Rabbit immunoglobulin, Medium: Culture medium alone, KP: Kisspeptin. Results are representative of 7 term placentas. * CM *vs*. CM-w/o KP(p<0.05–0.01 for MDA-MB-231 and p<0.05–0.001 for MCF-7), φ CM *vs*. medium (p<0.01–0.001 for MDA-MB-231 and p<0.05–0.01 for MCF-7), β CM *vs*. CM+anti-KP-10 (p<0.05–0.01 for MDA-MB-231 and p<0.05 for MCF-7), Φ CM-w/o KP *vs*. medium (p<0.05–0.001 for MDA-MB-231 and p<0.05 for MCF-7), ψ KP-10 *vs*. medium (p<0.05–0.01 for MDA-MB-231 and p<0.05–0.01 for MCF-7).

Potential inhibitory activity of placental KPs on proliferation of MDA-MB-231 and MCF-7 breast cancer cells was monitored over three days. According to the results, supernatants containing KPs significantly reduced proliferation of both cell lines compared to CM-w/o KP(p<0.05–0.001, depending on CM dilution and culture period) (Fig 3). Specific inhibitory action of placental KPs on proliferation of the cells, was shown by adding anti-KP-54 to the CM-treated cells, which resulted in significantly restored proliferation of both cell lines treated with 1:2 dilution of CM (p<0.05)(Fig 3A and 3B). Such activity of anti-KP-54 was due to the specific neutralization of soluble KPs, as addition of anti-KP-54 or the same concentration of normal rabbit IgG to the culture medium did not affect proliferation rate of the cells. 1:2 dilutions of CM exhibited anti-proliferative activity at all time intervals (p<0.05–0.001). Higher dilutions of CMs, however, gradually lost their anti-proliferative activity over time compared to the same dilutions of CM-w/o KP. Interestingly, 1:2 dilution of CM-w/o KP increased proliferation of MCF-7 cells in 24 and 48 hr culture periods (p<0.05) (Fig 3B), but such proliferation-stimulatory effect did not last for 72 hr. Contrary to MCF-7 cells, different dilutions of CM-w/o KP caused a significant inhibitory effect (p<0.05–0.001) on MDA-MB-231 cells which was more prominent at 48–72 h culture periods (Fig 3A). At the next step, we examined the effect of synthetic KP-10 on proliferation of breast cancer cells. In both cell types, 100 nM KP-10 exerted anti-proliferative activity in 24 and 48 hr culture periods (p<0.05–0.01), but no anti-proliferative activity was observed after 72 hr (Fig 3A and 3B).

### Placental kisspeptins reduces adhesive properties of MDA-MB-231 cells

**Fig 4:**
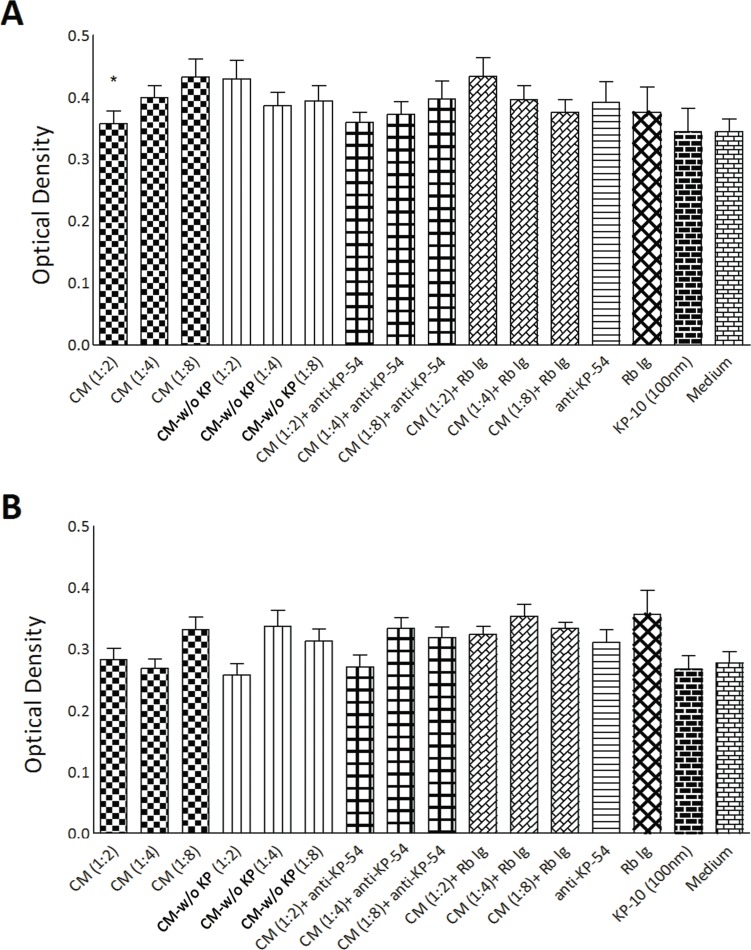
Effect of term placental kisspeptins on adhesion of MDA-MB-231 and MCF-7 breast cancer cells. MDA-MB-231 (A) and MCF-7 (B) cells were treated with different media as indicated in the Fig 3 for 150 min and their adhesion to fibronectin-coated plates was assessed by a colorimetric assay. Different dilutions from 1:2 to 1:8 were tested. CM: Term placenta conditioned medium containing KP, CM-w/o KP: KP-free CM, anti-KP: anti-Kp-54/Kp-145 antibody, Rb Ig: Rabbit immunoglobulin, Medium: Culture medium alone, KP: Kisspeptin. Results are representative of 11 term placentas. * CM *vs*. CM-w/o KP(p<0.05 for MDA-MB-231).

At the next step, potential effect of placental KPs on adhesion of breast cancer cells to extracellular matrix was examined. Cells were seeded in fibronectin-coated wells for 150 min and differentially treated with different media listed in proliferation assay. The level of attachment was then measured photometrically. Compared to CM-w/o KP, 1:2 dilution of CM significantly reduced adhesive properties of MDA-MB-231 cells (p<0.05)(Fig 4A). However, no significant effect was observed when MCF-7 cells were treated with CM. KP-10 exerted no significant effect on adhesion of both breast cancer cell lines.

### Placental kisspeptins potentiates invasiveness of MDA-MB-231 cells

**Fig 5:**
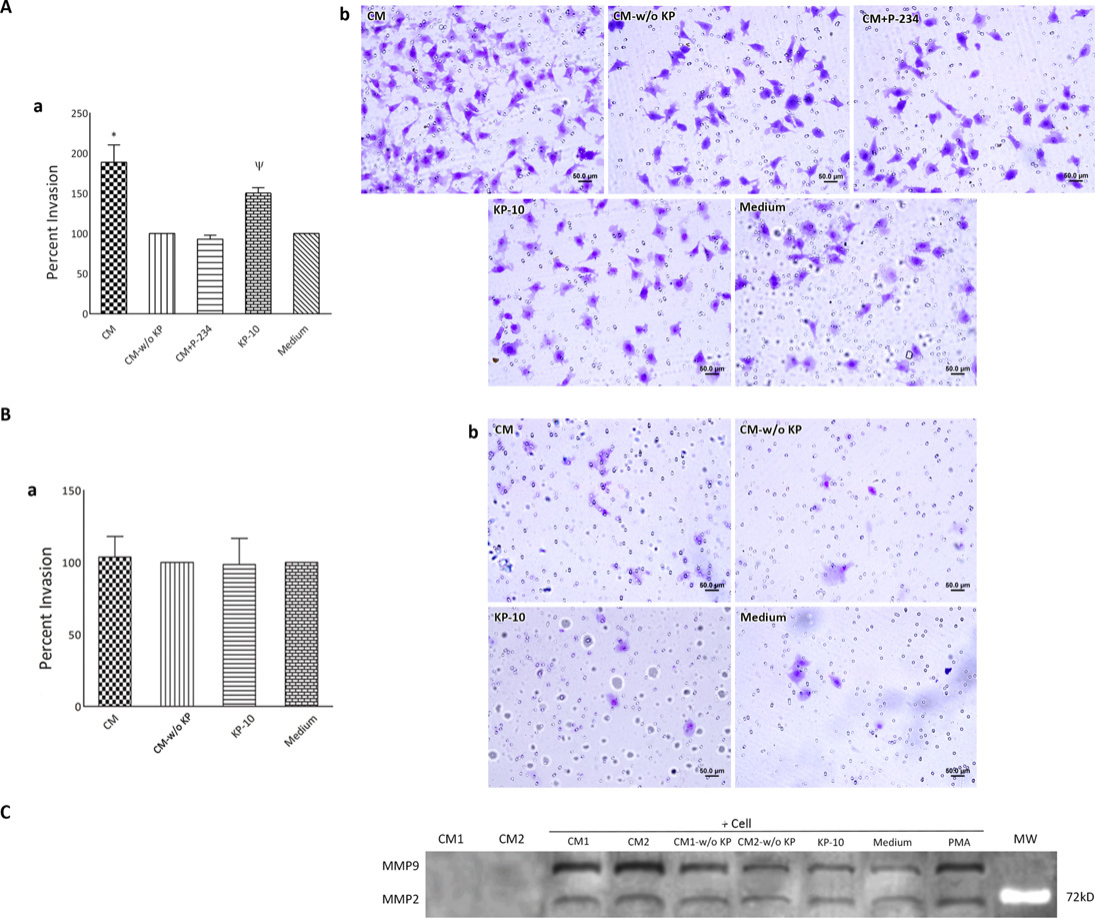
Effect of term placental kisspeptins on invasiveness of MDA-MB-231 and MCF-7 breast cancer cells. MDA-MB-231 (A) and MCF-7 (B) cells were treated with supernatants of cultured term placental explants containing KP (CM), CM plus 1 μM KISS1R antagonist; P-234, KP-free CM (CM-w/o KP), KP-10 or culture medium alone and their invasion toward chemo attractant (FCS) through Matrigel-coated filters was investigated. Invading cells was enumerated in 50 random fields (Ab and Bb) and expressed as percentage of corresponding controls (Aa and Ba). C) Gelatin zymographic analysis for matrix metalloproteinase (MMP) expression. MDA-MB-231 cells treated with CM released higher amounts of MMP2 and MMP9 compared to those treated with either CM-w/o KP. In parallel, cells treated with KP-10 showed slightly higher MMP9 activity in comparison to cultured in medium alone. Cells treated with Phorbol 12-myristate 13-acetate (PMA) served as positive control. CM failed to express detectable levels of MMP2 and MMP9. Results of two (denoted by numbers) out of four samples are shown. KP: Kisspeptin, MW: Molecular weight.

At the next step, we examined whether placental KPs alter invasive potential of MDA-MB-231 and MCF-7 cells using Matrigel invasion assay. Cells were seeded on Matrigel-coated filters and allowed to invade across the Matrigel towards FCS. To find possible effect of placental KPs on invasive properties of the cells, CM containing KPs was added to the cells at final dilution of 1:2 during culture period. Cells treated with CM-w/o KP served as negative control. We observed that MDA-MB-231 cells treated with CM exhibited significantly higher invasiveness compared to those treated with the same dilution of corresponding CM-w/o KP(p<0.001). Co-treatment of the cells with P-234 and CM normalized the rate of cell invasion to the levels observed in cells treated with CM-w/o KP (Fig 5Aa and 5Ab). In parallel, KP-10 significantly stimulated MDA-MB-231 cell invasion towards FCS (p<0.05) (Fig 5Aa and 5Ab). Neither CM nor KP-10 did significantly alter invasive property of MCF-7 cells (Fig 5Ba and 5Bb). To find out the basis of increased invasiveness of MDA-MB-231 cells following treatment with CM, gelatin zymography was performed. Accordingly, we found that MDA-MB-231 cells treated with CM released higher amounts of MMP2 and MMP9 compared to those treated with either CM-w/o KP. In parallel, cells treated with KP-10 showed slightly higher MMP9 activity in comparison to cultured in medium alone. Interestingly, supernatant of cultured term placental explants failed to express detectable levels of MMP2 and MMP9 activities (Fig 5C).

### Motility of MDA-MB-231 and MCF-7 cells is inversely affected by placental kisspeptins

**Fig 6:**
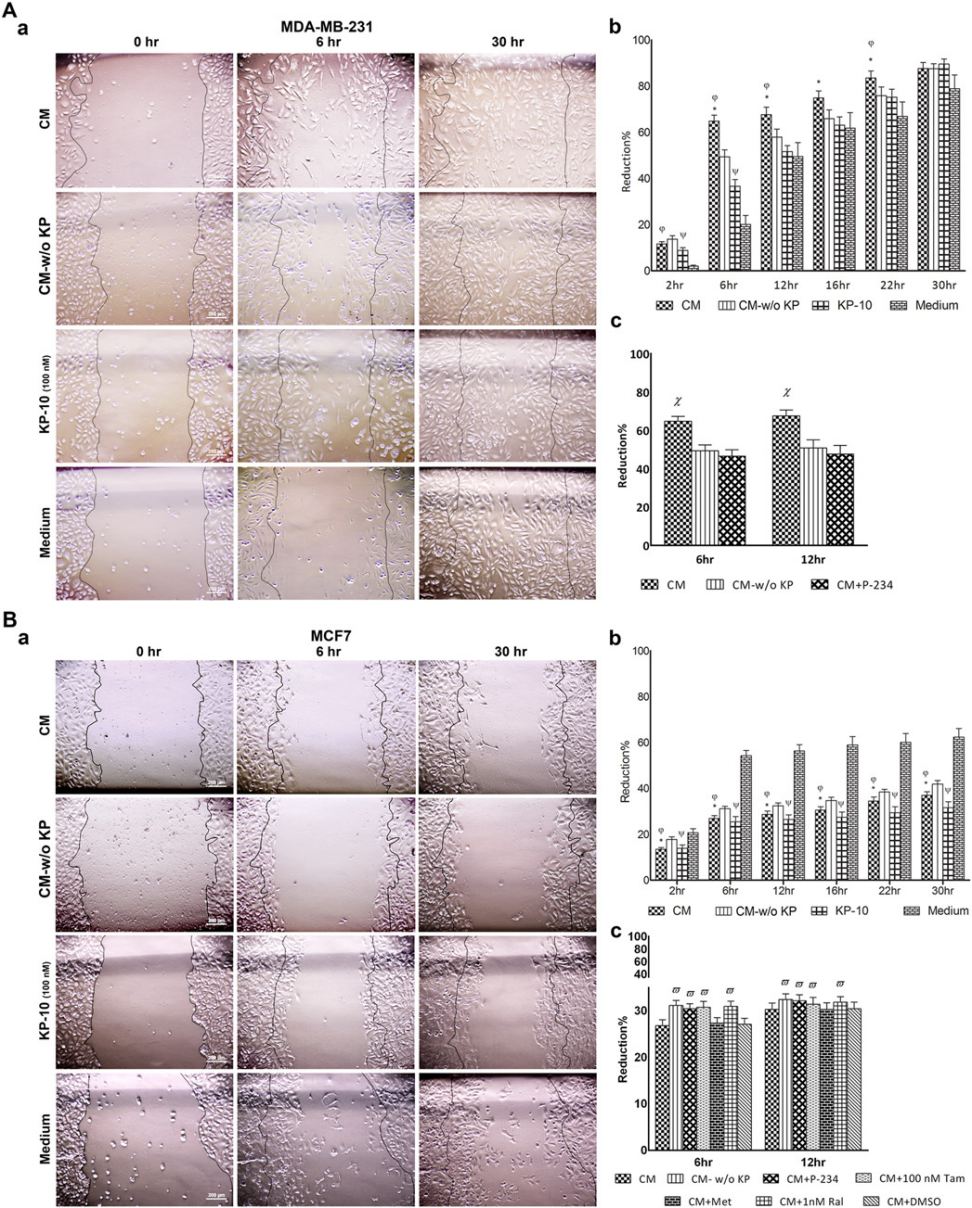
Effect of term placental kisspeptins on motility of MDA-MB-231 and MCF-7 breast cancer cells. MDA-MB-231 (A) and MCF-7 (B) cells were treated with supernatants of cultured term placental explants containing KP (CM), KP-free CM (CM-w/o KP), KP-10 or culture medium alone and their migration toward scratch was monitored during a period of 30 hr. Representative photographs are shown in Aa (MDA-MB-231) and Ba (MCF-7). Percent of reduction in scratched areas at each time point compared to initial time point was measured and analyzed using Image J software (Ab for MDA-MB-231 and Bb for MCF-7). Treatment with P-234 reversed the effect of CM on motility of MDA-MB-231 (Ac) and MCF-7 (Bc). Co-treatment with selective ER modulators inhibited the effect of CM on motility of MCF-7 cells (Bc). * CM *vs*. CM-w/o KP (p<0.05–0.001 for MDA-MB-231 and p<0.05–0.001), φ CM *vs*. medium (p<0.01–0.001 for MDA-MB-231 and p<0.001–0.0001 for MCF-7), ψ KP-10 *vs*. medium (p<0.05–0.01 for MDA-MB-231 and p<0.01–0.0001 for MCF-7),? CM *vs*. CM-w/o KP and CM+P-234 (p<0.05),? CM-w/o KP, CM+P-234, CM+100 nm Tam and CM+1 nm Ral *vs*. CM (p<0.05). Results are representative of 4 term placentas. Tam: Tamoxifen, Ral: Raloxifene.

Wound healing assay is a well-developed method to study directional cell migration *in vitro*. In this context, we conducted a tightly controlled *in vitro* scratch assay to investigate the effect of placental KPs on motility of breast cancer cell lines in two-dimensional culture system. Cell motility was monitored from initiation of culture and continued for 30 hr. To avoid any bias in determining motility rate due to possible variety in scratch diameters, reduction percent of scratch diameter in each well at different time intervals was calculated relative to the scratch diameter of the same well at time 0. We found that CM containing KPs significantly reduced motility of MCF-7 cells at all time points compared to CM-w/o KP(p<0.05–0.001)(Fig 6B). Supernatants of cultured placental explants whether contained KP (CM) or not (CM-w/o KP) exerted an intense inhibitory action on MCF-7 cell motility during entire culture period (p<0.001–0.0001). Such inhibitory activity was evident as early as 2 hr and lasted until the end of culture period (Fig 6). In parallel to the placental KPs, KP-10 also significantly reduced MCF-7 cell motility at all time intervals (p<0.01–0.001). Conversely, MDA-MB-231showed significantly stimulated motility after treatment with CM containing KPs (p<0.05–0.001) (Fig 6A). Such effect was more evident at 6 hr, after which stimulatory activity of placental KPs on cell motility was gradually subsided till 30 hr, when no statistical difference was found in cell motility of the cells treated with CM compared to those treated with CM-w/o KP. In contrast to MCF-7 cells, both placental explant culture supernatants containing KPs (CM) or lacking it (CM-w/o KP) intensely increased MDA-MB-231 cell motility (p<0.01–0.001) (Fig 6A). The most intensive stimulatory activity on motility of MDA-MB-231 cells was observed at 6 hr. Comparable to the action of placental KPs, KP-10 exerted considerable stimulatory activity on motility of MDA-MB-231 cells (p<0.05–0.01) (Fig 6A). The effects of CM on motility of breast cancer cells were reversed by P-234 (p<0.05) (Fig 6Ac and 6Bc). In parallel, co-treatment with selective ER modulators inhibited the effect of CM on motility of MCF-7 cells (p<0.05) (Fig 6Bc).

### Placental kisspeptins modulates pro-inflammatory cytokine production by breast cancer cells

**Fig 7:**
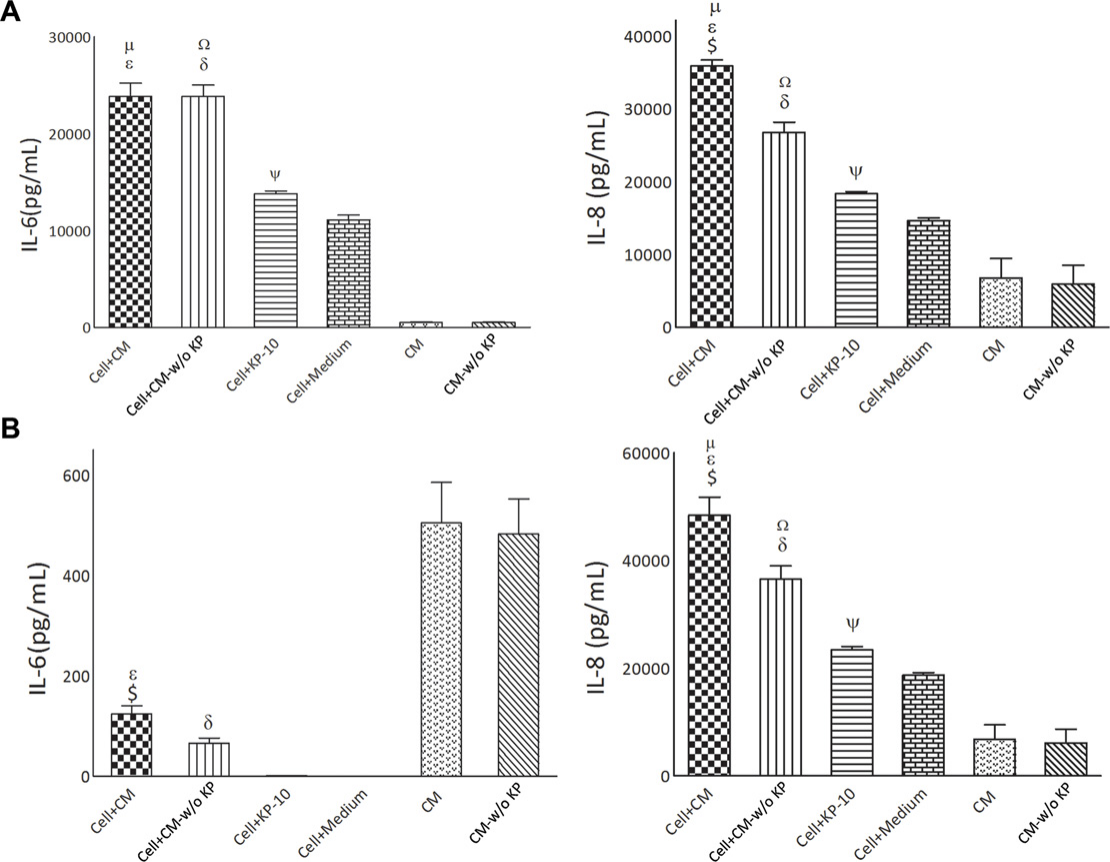
Effect of term placental kisspeptins on cytokine production by MDA-MB-231 and MCF-7 breast cancer cells. Concentrations of IL-6 and IL-8 were measured in culture supernatants of MDA-MB-231 (A) and MCF-7 (B) cells treated with CM, CM-w/o KP, KP-10 or culture medium. To exclude overlap of placental-derived cytokines with those produced by breast cancer cells, we also measured cytokine levels in CM and CM-w/o KP. $ Cell + CM *vs*. Cell + CM-w/o KP[(IL-6: p<0.05 for MCF-7), (IL-8: p<0.01 for MDA-MB-231 and p<0.01 for MCF-7)], ε Cell + CM *vs*. CM [(IL-6: p<0.001 for both breast cancer cells), (IL-8: p<0.01 for MDA-MB-231 and p<0.0001 for MCF-7)], μ Cell + CM *vs*. Cell + medium [(IL-6: p<0.05 for MDA-MB-231), (IL-8: p<0.05 for MDA-MB-231 and p<0.01 for MCF-7)], Ω Cell + CM-w/o KP *vs*. Cell + medium [(IL-6: p<0.05 for MDA-MB-231), (IL-8: p<0.05 for MDA-MB-231 and p<0.01 for MCF-7)], δ Cell + CM-w/o KP *vs*. CM-w/o KP[(IL-6: p<0.001 for MDA-MB-231, p<0.001 for MCF-7), (IL-8: p<0.01 for MDA-MB-231 and p<0.0001 for MCF-7)], ψ Cell+KP-10 *vs*. Cell + medium [(IL-6: p<0.05 for MDA-MB-231), (IL-8: p<0.05 for MDA-MB-231 and p<0.05 for MCF-7

IL-6 and IL-8 are among pro-inflammatory cytokines with profound effects on biology of cancer cells. We therefore investigated whether the production of these cytokines by breast cancer cells are affected by placental KPs. To this end, concentrations of these cytokines were measured in culture supernatants of breast cancer cells treated with CM, CM-w/o KP, KP-10 or culture medium. To exclude overlap of placental-derived cytokines with those produced by breast cancer cells, we also measured cytokine levels in CM and CM-w/o KP. To avoid any bias due to decomposition of placental cytokines during treatment period of breast cancer cells (30 hr), CM and CM-w/o KP were first incubated for 30 hr in CO2 incubator before measurement of cytokine levels. We observed that CM and CM-w/o KP contained considerable amount of IL-6 indicating production of this cytokine by placental villi. No detectable levels of IL-6 were found to be produced by MCF-7 cells. Concentration of IL-6 in supernatant of MCF-7 treated with either CM or CM-w/o KP was significantly lower compared to that in CM or CM-w/o KP, respectively (p<0.001) (Fig 7B). Compared to the cells treated with CM-w/o KP, there was higher concentration of IL-6 in cells treated with CM (p<0.05). KP-10 did not exert any significant effect on IL-6 production by MCF-7 cells (Fig 7B). In contrast to MCF-7 cells, MDA-MB-231 cells produced very high levels (at nanogram scale) of IL-6. Both CM and CM-w/o KP caused high production of IL-6 by MDA-MB-231 cells (p<0.001) (Fig 7A). A slight but significant increase in IL-6 production was observed when MDA-MB-231 cells were treated with KP-10 (p<0.05)(Fig 7A). Both CM and CM-w/o KP contained high amounts of IL-8. The level of IL-8 in supernatant of MDA-MB-231(p<0.01) and MCF-7 (p<0.0001) cells treated with CM was higher compared to that in CM (Fig 7A and 7B). Cells treated with CM produced more IL-8 compared to those treated with CM-w/o KP (p<0.01). KP-10 increased IL-8 production by MDA-MB-231(p<0.05) and MCF-7 (p<0.05) cells (Fig 7A and 7B).

## Discussion

Thus far, anti-cancer activity of KPs has been demonstrated in numerous cancers[27]. In case of breast cancer, however, there are rather limited and somehow conflicting data on KP-mediated modulation of cancer cell vital parameters including proliferation, migration, invasion and adhesion; mainly based on synthetic KP-10, the most active fragment of KPs. Here, we report for the first time the effects of the activity of placental KPs on proliferation, invasion, motility, adhesion and cytokine production in ER-negative and ER-positive breast cancer cells. Contrary to what was reported earlier[7], we showed that term placenta expresses KPs, mostly restricted to the syncytiotrophoblast surface of villi. In Western blot analyses, polyclonal antibody generated against KP-54, which is a part of KP-145, detected KP-145 in first trimester and term placental lysate and CM, but not other variants of KPs. Negative results for smaller fragments of KPs was not due to the nonreactivity of anti-KP-54 against these fragments, we showed that anti-KP-54 is able to detect purified KP-10 in Western blotting and its reactivity against KP-54 with the same methodology has already been documented[7]. This may be in part due to the rapid degradation of intracellular KP-54 because of the presence of PEST sequences predisposing KP-54 to proteosome degradation[28]. But the most probable scenario is that levels of intra- or extracellular KP-54 or smaller KP fragments are below the detection limit of western blotting as Bilban *et al*. [7] using MALDI-TOF showed that first trimester placenta conditioned medium contained KP-10, 13,14 as well as KP-54 in spite of failure to detect these variants in the same medium with Western blotting. Expression of KPs by term placenta in spite of very low level expression of MMPs and no/low invasiveness of trophoblasts indicates that KPs may be involved in other molecular mechanisms besides their role in invasion control.

Allthough *KISS1* mRNA is expressed by MDA-MB-231 and MCF-7 cells, no KISS1-specific signal was detected in Western blotting or immunofluorescent staining. Presumably, KP-145 associated with breast cancer cells undergoes rapid degradation by furin or prohormone convertases. This suggestion is supported by the fact that breast cancer tissues and cell lines including MDA-MB-231 and MCF-7 express elevated levels of furin and other members of the pro-protein convertase[29].

We next evaluated potential effect of placenta KPs on proliferation of MCF-7 and MDA-MB-231 breast cancer cells. Data on modulatory effect of KP-10 on breast cancer cell proliferation are limited and generally show no association between KP-10 stimulation and cancer cell growth. In a paper published by Kostadima *et al*.[30], no association of *KISS1* expression was found with genes that regulate cell-cycle and proliferation such as *HER2*, *VEGF* and *p53*. Indeed, it was shown that proliferation of breast epithelial or breast cancer cell lines including MCF-7 and MDA-MB-231, expressing the receptor endogenously or exogenously, was not affected by KP-10[30–32]. Our results clearly demonstrated that placental KPs markedly reduced proliferation of breast cancer cells in a dose- and time-dependent manner, regardless of ER status of cancer cells. This observation may be due to cumulative effects of different placental KPs fragments working in concert. Indeed, activity of the proteins is extensively influenced by their structure and it is highly probable that placental KPs with native conformation are more stable compared to synthetic polypeptides. In support of this hypothesis, our results showed that placental KPs have higher potency to block cell proliferation in general and retained its activity until 72 hr, when no anti-proliferative activity for KP-10 was observed.

In sharp contrast to MDA-MB-231 cells, conditioned medium lacking KPs increased proliferation of MCF-7 cells. Such reverse effects on cell proliferation can be interpreted in part by differential responsiveness of MCF-7 and MDA-MB-231 to the estradiol produced by placenta. It has been reported that 17β-estradiol inhibits MDA-MB-231 breast cancer cell growth by increasing BAX/BCL-2 and reducing pERK1/2[33], while it is mostly agreed that it promotes proliferation and cell-cycle progression of MCF-7 cells. More importantly, ER was shown to have differential cross-talk with activation protein 1 (AP1) transcription factors in these cells types; as it facilitates AP1 activity in MCF-7 cells, but inhibits their activity in such ER^-^cancer cells as MDA-MB-231 cells[34].

Next, we observed that CM and KP-10 significantly reduced motility of MCF-7 cells compared to CM-w/o KP. Notably, placental explant conditioned medium with or without KP-exerted an intense inhibitory action on MCF-7 cell motility. The opposite was the case for MDA-MB-231 cells, where CM-w/o KP and to a significantly higher degree CM increased MDA-MB-231 cell motility. Available data on placental manipulation of breast cancer cell migration and motility is mostly focused on first trimester placenta. In a recent work by Shochet *et al*. and contrary to what we found, it was shown that first trimester placental supernatants increased migration of ER^+^ breast cancer cells, MCF-7 and T47D, in scratch assay and that progesterone and E2 were significantly involved in mediating this effect[35]. The reason behind such discrepancy is not clear for us but two hypotheses could be put forth. First, despite availability of substantial information on the process of breast cancer metastasis, the role of ER signaling in this process remains poorly understood. Some studies proposed a negative effect of E2 on motility of ER^+^ breast cancer cells, while others showed a positive effect[34, 36, 37]. These diverging results may stem from dose-dependent action of E2 on cancer cell vital parameters[38] or from different methodologies used for assessment of cell motility. Notably, production of E2 by placenta is steadily increased throughout the gestation resulting in a totally different E2 concentration in supernatants of first trimester and term placental conditioned medium. Secondly, according to the recent findings, more than half of the genes that are expressed in the human placenta change their expression profile from the first to the third trimester of pregnancy indicating a substantial molecular rearrangement of placenta during development. Remarkably, genes involved in cell proliferation, cell differentiation and angiogenesis were up-regulated in the first compared to third trimester placenta[39]. In an elegant work conducted by Taylor *et al*., elegantly demonstrated that placental growth factor (PlGF) enhanced motility of MDA-MB-231 cells by mobilizing ERK1/2 phosphorylation, rearrangements of the actin cytoskeleton and stabilization of cytokeratin 19 and vimentin expression[40], a finding which supports our results on increased migration of MDA-MB-231 cells following treatment with placental supernatants. Interestingly, placental KPs exerted opposite effects on migration and invasiveness of MCF-7 and MDA-MB-231 cells. Metastasis is a complex process involving loss of cell-cell contact, invasion into neighboring tissues and spreading of tumor cells to the distant tissues. Despite well-documented studies showing anti-metastatic action of KISS1/KISSR signaling in numerous cancer types, the role of this system in breast cancer metastasis remained controversial. Some reports shows positive correlation between *KISS1* mRNA expression or KISS1R signaling and breast cancer progression and metastasis[15, 16], while the results of other reports indicate that KISS1 is required for metastasis suppression and maintenance of tumor dormancy[30, 41–43].To elucidate such discrepancy, Cvetkovic´*et al*. examined potential activity of KP-10 on estrogen receptor positive and negative cell lines and demonstrated that KP-10 stimulates invasion of estrogen receptor negative MDA-MB-231 breast cancer cells via transactivation of the epidermal growth factor receptor (EGFR). They showed that KP-10-enhanced invasiveness of MDA-MB-231 cells was ablated by exogenous expression of ERalpha. In sharp contrast with ER negative cell line, neither transactivation of EGFR nor stimulation of invasiveness was observed when ER positive MCF-7 and T47D breast cancer cells were treated with KP-10. This observation suggests that ER expression status may be a basis for whether or not invasiveness and migration of breast cancer cells are affected by KISS1/KISS1R system [31]. In line with this findings, Zajac *et al*. showed that KP-10 stimulated invasion and increased MMP9 activity in MDA-MB-231 breast cancer cells[44]. In order to test the effect of ER signaling on reduced motility of MCF-7 cells following treatment with CM, we treated the cells with selective ER modulators, Tamoxifen and Raloxifene, and found that such treatments could significantly reverse the effect of CM on cell motility to the levels observed in cells treated with CM-w/o KP. These results are in line with the previous reports showing the influence of ER signaling on KP modulation of cell migration. Our findings also revealed that in parallel to KP-10, placental KPs stimulated migration and invasion of KISS1^+^/KISS1R^+^/ER^-^ MDA-MB-231 cells, a finding which was concomitant with secretion of MMP9 and MMP2. Co-treatment of MDA-MB-231 cells with KISS1R antagonist, P-234, and CM blocked CM-mediated increased invasiveness and motility of these cells indicating that KISS1R is involved in regulating the process of cell invasion following treatment of the cells with CM.

Interestingly, placental KPs markedly reduced migration potential of KISS1^+^/KISSR^+^/ER^+^ MCF-7 cells which was reversed by P-234. These findings reinforce the concept that it is ER status of breast epithelial cells that critically regulates the ability of KISS1R signaling to stimulate invasiveness. In fact, Olbrich *et al*. showed that bone-directed migration of MCF-7 cells is inhibited by KP-10 through down-regulating the expression of chemokine receptor CXCR4 and its ligand, stromal derived factor (SDF)-1[45]. We found that placental KPs significantly reduced adhesive properties of MDA-MB-231 cells, but exerted no effect on MCF-7 adhesion. This finding supports our results on increased invasiveness of MDA-MB-231 cells following treatment with CM containing KPs. KP-10, however, did not affect adhesiveness of both cells types. According to previous reports, KP-10 does not affect overall rate of cell adhesion to ECM and regulates events downstream of cell–matrix adhesion, perhaps involving cytoskeletal re-organization[14].

Pro-inflammatory cytokines play an important role in breast cancer biology. Therefore, to find a mechanistic basis of placental KP-modulation of breast cancer cells vital parameters, we next examined whether or not production of IL-6 and IL-8 are affected by placental KPs. We did not find any report connecting KPs and production of pro-inflammatory cytokines. Whether or not differential effect of placental KP on pro-inflammatory cytokine production by MCF-7 and MDA-MB-231 cells is under control of ER signaling, is not clear at present. But there are some reports showing that ER impairs IL-6 expression by preventing protein binding on the NF-κB site[46], a finding which is in line with our results for MCF-7. We showed that CM, compared to CM-w/o KP, caused a significant increase in IL-6 concentration in supernatant of treated MCF-7 cells showing the positive effect of placental KPs in IL-6 levels. This finding may be a basis for anti-proliferative effect of placental KPs as it was shown that direct application of IL-6 on breast cancer cells inhibits proliferation in estrogen receptor positive cells[47, 48]. Interestingly, proliferation of ER^-^ breast cancer cells are not affected by IL-6 [47] implying IL-6-independent modulation of ER^-^ MDA-MB-231 cells proliferation. Moreover we found that IL-6 expression by MDA-MB-231 cells is not affected by placental KPs. We also observed that placental KPs increased IL-8 production by MDA-MB-231 and MCF-7 cells. Interestingly, the invasion potential of ER^-^ breast cancer cells is associated at least in part with expression of IL-8 [49]. This data is in line with stimulatory action of placental KPs on invasive capacity of MDA-MB-231 cells. On the other hand, this cytokine considerably induces migration of MDA-MB-231 cells, while inhibits migration of MCF-7 cells [50] Increased migration of MDA-MB-231 cells and decreased migration of MCF-7 cells following treatment with placental KPs may be in part interpreted by stimulatory activity of placental KPs on IL-8 production by these breast cancer cell types.

Collectively, here we reported the results of comprehensive analyses of placental KP-modulation of breast cancer cells. The data presented here clearly showed that placental KP differentially affected proliferation, invasion, adhesion, and motility of ER-negative and ER-positive breast cancer cells possibly by modulation of pro-inflammatory cytokine production and that in most instances such effects were in parallel to and in some instances stronger than what was exerted by synthetic KP-10. Our results suggest that placental factors may reduce breast cancer progression depending on ER status of cancerous cells and support the concept that it is the ER status of mammary cells that dictates whether or not KISS1R may be a novel clinical target for treating breast cancer metastasis.

## Conclusion

Taken together, our observations suggest that placental KPs differentially modulate vital parameters of estrogen receptor-positive and -negative BC cells possibly through modulation of pro-inflammatory cytokine production.
